# The Effects of a Ketogenic Diet on Patients with Dihydrolipoamide Dehydrogenase Deficiency

**DOI:** 10.3390/nu13103523

**Published:** 2021-10-07

**Authors:** Orna Staretz-Chacham, Ben Pode-Shakked, Eyal Kristal, Smadar Yaala Abraham, Keren Porper, Ohad Wormser, Ilan Shelef, Yair Anikster

**Affiliations:** 1 Metabolic Clinic, Soroka Medical Center, Beer Sheva 8480101, Israel; 2 Faculty of Health Sciences, Ben-Gurion University, Beer Sheva 8410501, Israel; eyalkristal@gmail.com (E.K.); shelef@bgu.ac.il (I.S.); 3 Metabolic Disease Unit, Edmond and Lily Safra Children’s Hospital, Sheba Medical Center, Tel-Hashomer, Ramat Gan 5262000, Israel; ben_pode@hotmail.com (B.P.-S.); Smadar.Abraham@sheba.health.gov.il (S.Y.A.); kerenporper83@gmail.com (K.P.); Yair.Anikster@sheba.health.gov.il (Y.A.); 4 Sackler Faculty of Medicine, Tel-Aviv University, Tel-Aviv 6997801, Israel; 5 Pediatric Ambulatory Day Unit, Soroka Medical Center, Beer Sheva 8480101, Israel; 6 The Morris Kahn Laboratory of Human Genetics, National Institute for Biotechnology in the Negev, Faculty of Health Sciences, Ben Gurion University of the Negev, Beer Sheva 8410501, Israel; wormser@post.bgu.ac.il; 7 Department of Radiology, Soroka University Medical Center, Beer Sheva 8480101, Israel; 8 The Wohl Institute for Translational Medicine, Sheba Medical Center, Tel-Hashomer, Ramat Gan 5262000, Israel

**Keywords:** dihydrolipoamide dehydrogenase (DLD), pyruvate dehydrogenase complex (PDHC), ketogenic diet

## Abstract

Background: Dihydrolipoamide dehydrogenase (DLD lipoamide dehydrogenase, the E3 subunit of the pyruvate dehydrogenase complex (PDHC)) is the third catalytic enzyme of the PDHC, which converts pyruvate to acetyl-CoA catalyzed with the introduction of acetyl-CoA to the tricyclic acid (TCA) cycle. In humans, PDHC plays an important role in maintaining glycose homeostasis in an aerobic, energy-generating process. Inherited DLD-E3 deficiency, caused by the pathogenic variants in DLD, leads to variable presentations and courses of illness, ranging from myopathy, recurrent episodes of liver disease and vomiting, to Leigh disease and early death. Currently, there is no consensus on treatment guidelines, although one suggested solution is a ketogenic diet (KD). Objective: To describe the use and effects of KD in patients with DLD-E3 deficiency, compared to the standard treatment. Results: Sixteen patients were included. Of these, eight were from a historical cohort, and of the other eight, four were on a partial KD. All patients were homozygous for the D479V (or D444V, which corresponds to the mutated mature protein without the mitochondrial targeting sequence) pathogenic variant in DLD. The treatment with partial KD was found to improve patient survival. However, compared to a historical cohort, the patients’ quality of life (QOL) was not significantly improved. Conclusions: The use of KD offers an advantage regarding survival; however, there is no significant improvement in QOL.

## 1. Introduction

Dihydrolipoamide Dehydrogenase (DLD), also known as the E3 subunit of the pyruvate dehydrogenase complex, EC 1.6.4.3, is the third catalytic enzyme of the pyruvate dehydrogenase complex (PDHC), which is a multifunctional, mitochondrial matrix enzyme. DLD-E3 is a flavoprotein shared by a number of mitochondrial enzymatic complexes: AKGDHc (α-ketoglutarate dehydrogenase complex), BCKDHc (branched chain a-ketoacid dehydrogenase) and pyruvate dehydrogenase complex (PDHc) [1,2]. The main role of PDHC is to convert pyruvate to acetyl-CoA catalyzed by the oxidative decarboxylation of pyruvate and the formation of acetyl-CoA, CO2 and NADH with the introduction of acetyl-CoA to the tricarboxylic acid (TCA) cycle. In humans, PDHC acts as the gatekeeper, maintaining glucose homeostasis during the eating and fasting states as part of the metabolism of pyruvate [3]. This is an aerobic, energy-generating pathway [1].

Pathogenic variants in the DLD gene are shown to cause DLD-E3 deficiency. This disorder manifests with a wide spectrum of presentations and courses of illness, ranging from severe neonatal disorders characterized by profound intermittent metabolic acidosis, neurological impairments in the form of Leigh disease and fatal outcomes to myopathy, or recurrent episodes of liver disease and vomiting. Different pathogenic variants are linked with the different presentations, for example: severe neonatal presentation with a cognitive impairment was reported with K72E/P488L [4], I353T and G136del [5], Y35Xins/R495G [6], E375K/p.M361V [7], R482G [8] and the D479V [9] variants reported herein; liver involvement was reported with G229C [10,11,12,13] and the myopathic form with I40Lfs*4/G461E [14], I480M [15], etc. (Supplementary Table S1). Three main types of presentation were reported. Firstly, there was the early onset that manifested in early infancy, as early as the first hours of life, with severe lactic acidosis and profound hypotonia. The affected infants most commonly died within the first few years of life during a recurrent metabolic decompensation, and those who lived beyond the first two to three years frequently exhibited severe failures to thrive and residual neurologic deficits (intellectual disability, spasticity, ataxia, and seizures) [16]. The second form of presentation is the late-onset milder form of this disease or the liver involvement characterized by episodic vomiting, metabolic acidosis, hyperammonemia, elevated liver enzymes and hypotonia with no residual deficits between episodes [12]. The acute metabolic decompensation can lead to liver failure and result in death. The third form of presentation is the myopathic form characterized by cramps, muscle weakness, and exertional fatigue with elevated creatine kinase [14].

To date, there is no definitive treatment, nor consensus guidelines or recommended therapy. One of the suggested treatments is a ketogenic diet (KD), a high-fat, low-carbohydrate diet, which mimics a metabolic state of prolonged starvation, forcing the body to utilize fat as the primary source of energy instead of glucose [17]. KD is utilized as part of the treatment of paediatric patients with refractory seizures and the treatment of choice for certain inborn malfunctions in metabolism [18,19] and should be considered for the treatment of PDHC.

In PDHC deficiency, the glycolytic end product, pyruvate, is not optimally metabolized through the TCA cycle, leading to the increased production of lactate and the impaired production of adenosine triphosphatase (ATP) via the mitochondrial respiratory chain [19,20]. As a result of increased fatty acid oxidation in the liver, there is an increase in Acetyl CoA levels. These levels are compressed to generate the ketone bodies (acetate and beta-hydroxybutyrate) and then transferred from the liver to the cells through the bloodstream, serving as an alternative fuel, instead of glucose for the brain [21]. Ketosis is considered to improve lactate levels in PDHC [19,22]. In this case, the ketogenic diet would be expected to lower the lactatemia with an improved survival and quality of life in patients with DLD-E3.

The KD is a restrictive diet which follows strict guidelines. There are four different versions of the KD: the traditional classic ketogenic diet based on a strict ketogenic ratio, calculated by mass (grams), between the fat and protein, and fat and carbohydrate (3:1, 4:1, respectively). Higher ratios result in greater degrees of ketosis and a better efficacy. In a 4:1 ratio, there are 4 g of fat for every 1 g of protein and carbohydrate combined. Protein is provided to meet the dietary reference intake, which is approximately 1 g per kilogram of body weight and carbohydrates complete the remaining allowance of the ratio (90% of calories coming from fat, 8% from protein and 2% from carbohydrate) [23]. KetoCal (Nutricia, Gaithersburg, MD, USA) is a 4:1 ratio, (fat: carbohydrates and protein in grams) and a nutritionally complete, formula-based powder that can be used as a meal substitute for infants and children [23]. The initiation of the diet usually requires hospitalization and weighing individual food items. For a less restrictive KD with a lower ratio, the KetoCal can be combined with a standard infant formula [23,24].

The medium-chain triglyceride (MCT) diet based on MCT oil secreted from coconut oil, is thought to stimulate the hepatic production of ketone bodies [25]. The MCT diet contains 60% calories coming from MCT oil as the main source, 11% from polyunsaturated sources, 10% from protein and 19% from carbohydrates. This results in a ratio of fat to non-fat of approximately 1:1, allowing more carbohydrates in the diet compared to the classic KD [23,25].

The Modified Atkins Diet and a diet with a Low Glycemic Index are considered less restrictive diets and usually administered for older children. Both versions result in an approximate ratio of 1:1(fat to nonfat), with 60–65% of calories coming from fat, 30% from protein, and 5–10% from carbohydrates [26,27]. The initiation of this diet does not require hospitalization, nor weighing individual food items.

Despite the efficacy of KD in patients with epilepsy and certain metabolic diseases, such as GLUT1 deficiency [28] and PDHC deficiency [22,29,30,31], many patients find it very difficult to persist with a prolonged restrictive diet like the ketogenic diet, especially when the treatment is for life [32].

So far, based on the previous experience of KD for PDHC as reported in the literature, seven patients diagnosed with DLD-E3 deficiency were treated with KD, five of them had no clinical or biochemical benefits, of these the conditions of two patients worsened and the other two improved clinically [7,33,34,35].

Based on the above reports and the severity of the disease in the current mutation, we hypothesized that a less restrictive partial KD would improve the patients’ quality of life while still remaining effective and would ameliorate the survival of these patients.

In this paper we report a novel approach of a less restrictive partial KD for patients diagnosed with DLD-E3 deficiency in a single medical center, all homozygous to the same mutation, which harbors a fatal Leigh disease phenotype.

## 2. Materials and Methods

Every patient born at the Soroka Medical Center between 2016 and 2020 and diagnosed with DLD-E3 due to homozygosity of the D479V pathogenic variant in the DLD gene (NM_000108.5) was offered a KD as detailed below.

We compared DLD patients born between 2016 and 2020 to a historical cohort of patients born between 2000 and 2016, focusing on data regarding the survival and quality of life, by measurements of development as well as imaging studies.

The study was conducted according to the guidelines of the Declaration of Helsinki, and approved by the Institutional Review Board (IRB) of the Soroka Medical Center (approval number SOR-0159-19). The need for individual written informed consent was waived as the study was based on questionnaires filled out by the attending physicians, and the paper does not contain identifiable personal details nor patient photos.

### 2.1. Dietary Intervention

#### 2.1.1. Partial Ketogenic Diet

The patients on a partial KD received KetoCal (Nutricia, Gaithersburg, MD, USA), a high-fat content formula (based on 4:1 ketogenic ratio) combined with a based milk standard infant formula, as part of their diet, with 60–70% of the total energy coming from fat. Protein was provided to meet infant dietary reference intake and above (2.5–3.5 g/kg) according to their nutritional needs. (Dietary details presented in Table 1).

#### 2.1.2. Parenteral Nutrition

All patients (regardless of KD) received lipofundin (B. Braun Melsungen AG, Melsungen, Germany) or SMOFlipid emulsion (Fresenius Kabi AB, Uppsala, Sweden) of up to 4 g/kg/day during a crisis as a supply of energy, and essential fatty acids and omega-3 fatty acids, as part of a parenteral nutrition regimen when oral or enteral nutrition was impossible, insufficient or contra-indicated. At the same time, hepatic enzymes were monitored. 

### 2.2. Imaging Studies

Brain magnetic resonance imaging (MRI) was performed as a clinical study in all treated by KD cases. Standard clinical protocols were used including T1, T2, and T1 fluid-attenuated inversion recovery and diffusion-weighted imaging. All images were reviewed by a pediatric neuroradiologist.

### 2.3. Biochemical Studies

Biochemical tests, including serum liver transaminases, serum lactate levels and venous blood gases, were periodically obtained as part of the clinical investigations, and performed by the Clinical Laboratory of the Soroka Medical Center.

## 3. Results

During the study period, eight patients were diagnosed as homozygous to D479V in the DLD gene, and four of them were treated with a ketogenic diet, and prescribed with additional sodium bicarbonate as needed. All patients treated with a partial KD underwent the insertion of a percutaneous gastrostomy (PEG), and one had a Nissen funduplication procedure. Patients treated with a partial ketogenic diet were fed orally or by PEG according to their choice. The other four were only treated with sodium bicarbonate, according to their parents’ decisions.

The historical cohort included eight patients diagnosed as homozygous to D479V in the DLD gene born between 2000–2016. Of the eight patients in the historical cohort, only two (25%) survived beyond 3.5 years with a weight of less than 10 kg (patients 9,10, Table 2), and all were severely encephalopathic and did not reach any developmental milestones (Table 3).

None of the patients who received a less restrictive partial KD had a worsened condition with the treatment; however, all patients had severe developmental delays (Table 3), with recurrent hospital admissions due to metabolic crises with different triggers, such as intercurrent infections. None of the patients started walking independently nor with assistance. None of the patients started talking, other than babbling.

Of note, while all patients were homozygous to the same mutation, their presentations were somewhat variable with regard to the severity of the disease and the age of death (Table 2 and Table 3). Nevertheless, while several patients died early and others survived, all patients in our cohort showed a very severe phenotype, as opposed to, for instance, previously reported patients harboring the G229C variant (Supplementary Table S1), further supporting the genotype–phenotype correlations in this disorder.

Only three patients (19%) of the whole cohort did not have any episodes of hypoglycemia, and the others had recurrent episodes during the metabolic crises. Only four patients (25%) had liver disease with elevated transaminases during metabolic crises (Table 3).

Four patients had urine organic profiles that all revealed elevated 2-OH-Butyrate and 3-OH-Butyrate > Acetoacetate (Table 4).

**Figure 1:**
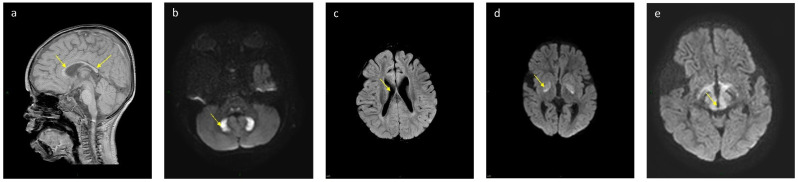
Representative Brain MRIs: (**a**) partial agenesis of corpus callosum. (**b**) DWI restriction of dentate nucleus. (**c**) moderate ventriculomegaly. (**d**) 2-DWI restriction of globus pallidum. (**e**) 2-DWI restriction of midbrain.

Six patients underwent brain imaging by magnetic resonance imaging (MRI) (patients 6, 7, 11, 12, 13, 16; of these, patients 11 and 13 were on regular diet) which revealed the partial agenesis of corpus callosum with a restriction of the dentate nucleus, moderate ventriculomegaly, the restriction of globus pallidum and of the midbrain (Figure 1), with no difference between those on PD to those on a regular diet (Table 5).

## 4. Discussion

DLD-E3 is a rare disorder with a variable spectrum of presentation and course of disease. We present a cohort homozygous to the same severe mutation with a devastating disease. Currently, there is neither a definitive treatment, nor consensus management guidelines for patients with DLD-E3 deficiency.

All the patients in our cohort were homozygous to the p.D479V (or p.D444V) [8] mutation in the DLD gene. So far, there is only one previous report of a patient homozygous to this mutation in the literature [9]. Additionally, so far only a few dozen [36] patients with DLD-E3 are reported worldwide (their clinical and molecular characteristics are summarized in Supplementary Table S1), and we add here a cohort of an additional 16 patients.

Shany et al. compared the current mutation to the G229C mutation, which was reported in recurrent episodic liver failure, and found that, although the DLD-E3 residual activity in the muscle homogenate was higher in the current mutation than in G229C, the multi-enzymatic complex activity in the current mutation was undetectable compared to the G229C with a higher residual activity. This most likely resulted from the location of the discussed mutation in the interface domain which led to the impaired stabilization of the PDHC complex, which was more important than the DLD-E3 for the activity, and therefore led to severe neurodegenerative disease [9].

Ambrus et al. found that the selected pathogenic variants of DLD-E3, including the D444V, produced elevated amounts of reactive oxygen species (ROS) that led to mitochondrial oxidative stress, resulting in brain damage. In addition, certain pathogenic variants will also lose their affinity for the E2 component of the PDHc, whilst a comparison with the PDHC showed only a significant ROS elevation in vitro [37]. The above described process oxidatively damaged the LA-cofactors KGDHc and PDHc, in the case of D444V, in human homozygous fibroblasts [38]. These could partially explain why KD that was built on lipids did not yield an improvement as expected in patients with DLD-E3 mutations.

Most of our patients in the past had a severe fatal course of the disease with early death, within 3 months in four patients of the historical cohort. The patients who were treated with a less restrictive partial KD in our study, also presented similar to these patients at birth, at least one of them presented two hours after birth with seizures due to hypoglycemia and acidosis; however, they did not succumb and survived the perinatal crisis, most likely due to close monitoring and mainly with the addition of fats in the diet and the frequent treatment with sodium bicarbonate. The addition of fats in the forms of lipofundin (B. Braun Melsungen AG, Melsungen, Germany) or SMOFlipid emulsion (Fresenius Kabi AB, Uppsala, Sweden) up to 4 g/kg/day changed the disease progression during the crisis in these severely affected patients, with a significant improvement following the introduction or increment of the fat amount.

Additionally, in recent years the close monitoring of these patients with an improved supportive therapy also added to the embellished survival rate, although the quality of life did not significantly improve. In this study we found that even if a patient achieves delayed milestones and says a few words, with time they unfortunately deteriorate to chronic encephalopathy, as can be seen in the description of patients (pt.7) (Table 2).

As can be noted in Table 4, the lactate–pyruvate ratio in eight patients was >20 which could be observed, either by taking after feeds, by improved control or even as an unreliable measure. No correlation to the diet was noted.

Brain imaging studies demonstrated that, even with the strict management and partial PD, patients 8, 11, and 13 all showed the severe changes consistent with Leigh disease. Additionally, when comparing these exams to brain MRIs of patients 12 and 14, who were on regular diets due to their parents’ preferences, no significant differences were seen.

One of the major issues to consider was the safety of treatment and, although the sample is small, none of our patients deteriorated under this regimen, nor developed any adverse events. Our findings were supported by a few studies indicating that the KD was an efficient and safe treatment during childhood. Its adverse effects, although common, were very mild [39,40,41]. Furthermore, more palatable but related diets, such as the modified Atkins diet (MAD) or the less restrictive KD, like the one evaluated in our study, were associated with fewer adverse effects [42,43,44]. However, these should be implemented under careful medical supervision.

The strength of our study is a cohort of 16 patients all homozygous to the same mutation of a rare disease, with only about twice as many [36] such patients reported in the literature to date. A limitation of the study is the lack of formal cognitive tests, although, due to the severity of the phenotype, these could not be performed.

As mentioned above, the administration of a less restrictive partial KD prolongs life; however, the quality of life does not seem to significantly improve, and even if there is a partial gaining of milestones, the patients eventually lose their acquired abilities. Thus, it is probable that the prenatal damage is so significant that, even with close monitoring and a specific diet, we cannot currently overcome it.

## 5. Conclusions

Our results show that while the treatment of patients with DLD-E3 deficiency with a partial KD improves survival (Table 2), the quality of life is not improved. Thus, we need to consider whether a stricter diet might have an improved quality of life. Another important consideration should be whether, with such a difficult regimen and no supportive results, this treatment should be offered to families. Additionally, we should consider future therapeutic measures such as vitamin supplementation, for example riboflavin as previously recommended [14], the supplementation of triheptanoin, or a targeted gene therapy.

## Figures and Tables

**Table 1:** Details of ketogenic diet for each patient.

Patient No.	Age *	Weight * (Kg)	Height * (cm)	Kcal	Kcal/Kg	Protein	Protein/Kg	%Fat	Ketogenic Ratio
12	3 y 3 m	7.900	79.8	1284	162.5	27.7	3.5	67.9	1:1.06
7	4 y 2 m	12.55	99	1362	108.5	41.8	3.3	61.5	1:1.4
16	3 y	7.940	79	923.2	116.2	20.1	2.53	63.5	1:1.29
6	5 y	10.900	95	1027	94.2	32.1	2.94	61.5	1:1.4

* All data is in current status.

**Table 2:** Demographic and clinical characteristics of patients with dihydrolipoamide dehydrogenase deficiency. Patients treated with partial ketogenic diet appear in bold.

Pt. No.	Gestational Age	Gender	Current Age/Death * (d/m/y)	First Presentation	Age at First Presentation	Treatment	No. of Hospitalizations (Per Year)
1 ^h^	33 + 2 weeks	F	* 37 m	Metabolic acidosis	Birth	Started Ketocal at 2 y	2–5
2 ^h^	Term/SGA	F	* 29 m	Metabolic acidosis	Birth	Bicarbonate in crisis	2
3 ^h^	Term/SGA	M	* 3 d	Metabolic acidosis	Birth	Fluids	-
4 ^h^	Term	M	* 4 d	Metabolic acidosis	2 d	Fluids	-
5 ^h^	Term	F	* 3 m	Metabolic acidosis	Birth	Fluids	1
**^k^ 6**	**Late Preterm**	**M**	**4.9 y**	**Metabolic acidosis and hypoglycemia**	**1 d**	**Partial ketogenic diet and Bicarbonate**	**2**
**^k^ 7**	**Term**	**M**	**4.1 y**	**Metabolic acidosis and hypoglycemia**	**1.5 d**	**Partial ketogenic diet and Bicarbonate**	**1–6**
8 ^h^	Term	M	* 3 d	Metabolic acidosis	Birth	-	-
9 ^h^	Term	F	16 y	Metabolic acidosis and cutis	Birth	Canola oil	1–2
10 ^h^	Term/SGA	M	14.2 y	Metabolic acidosis and hypertonus	Birth	-	2–5
11	Term	M	4.3 y	Metabolic acidosis	Birth	Bicarbonate	1
**^k^ 12**	**Term**	**F**	**3.2 y**	**Metabolic acidosis**	**Birth**	**Partial ketogenic diet and Bicarbonate**	**4–6**
13	Term	M	3.5 y	Metabolic acidosis	18 m	Bicarbonate	4
14	Term/SGA	F	1.5 y	Metabolic acidosis	Birth	Bicarbonate	4
15	Term/SGA	F	1.5 y	Metabolic acidosis	Birth	Bicarbonate	4
**^k^ 16**	**Term**	**M**	**2.8 y**	**Metabolic acidosis and seizures**	**Birth**	**Partial ketogenic diet and Bicarbonate**	**2–3**

SGA, small for gestational age. * Age of death. ^h^ Historical cohort. ^K^ Ketogenic Diet.

**Table 3:** Further clinical characteristics in patients with dihydrolipoamide dehydrogenase deficiency: hypoglycemic events and developmental milestones.

Pt. No.	Current Age/Death * (D)-(d/m/y)	GER	Liver Disease	Hypoglycemia	Most Advanced Development	Seizure	DD/ID/ PMD
1 ^h^	* 37 m	No	No	No	Smiled	No	Severe
2 ^h^	* 29 m	No	No	Repeated	Rolled both sides, said syllables	No	Severe
3 ^h^	* 3 d	No	No	Yes	NA	No	NA
4 ^h^	* 4 d	No	No	Yes	NA	No	NA
5 ^h^	* 3 m	No	No	Yes	Hypertonus, no spontaneous smiles	No	Severe
^k^ 6	4.9 y	No	During crisis	Yes	Sat, rolled, said five words but regressed at 46 months and now does not say a word and no eye contact	No	Severe
^k^ 7	4.1 y	Severe	During crisis	Yes	Rolls both sides, says syllables, sat with support, said 7 words but regressed at 48 months and now does not say a word and only smiles	Yes	Severe
8 ^h^	* 3 d	No	No	No	NA	No	NA
9 ^h^	16 y	No	Mild during crisis	No	Severe spastic PMD, no communication, mastication movements	No	Severe
10 ^h^	14.2 y	No	During crisis	During crisis	Severe spastic PMD, no communication	No	Severe
11	4.3 y	No	No	During crisis	Smiles, says syllables, rolls and sits by himself	No	Moderate to severe
^k^ 12	3.2 y	No	No	During crisis	Smiles; says syllables, rolls both sides, transfers objects, holds her bottle, sits with support	Yes	Moderate to severe
13	3.5 y	No	No	No	Wandering eyes, no communication	Yes	Severe
14	1.5 y	No	No	During crisis	Smiles, rolls both sides, says syllables	No	Moderate to severe
15	1.5 y	No	No	During crisis	Smiles, rolls both sides, says syllables, sits with support	No	Moderate to severe
^k^ 16	2.8 y	No	No	No	Smiles, rolls both sides, says syllables	Yes	Severe

DD, developmental delay; GER, gastroesophageal reflux; ID, intellectual disability; NA, not available; PMD, psychomotor delay; * Age of death, ^h^ Historical cohort, ^k^ Ketogenic Diet.

**Table 4:** Biochemical studies in patients with dihydrolipoamide dehydrogenase deficiency.

Pt. No.	Urine Organic Acids	Highest Lactate (Norm: 0.5–2.2 mmol/L)	Pyruvate (Norm: 0.03–0.08 mmol/L)	Lactate/Pyruvate Ratio
1 ^h^	NA	19	NA	NA
2 ^h^	Elevated lactate, 2-OH-Butyrate Elevated 3-OH-Butyrate > Acetoacetate	7.6	1.98	3.88
3 ^h^	NA	17	2	8.5
4 ^h^	NA	18	1	18
5 ^h^	NA	5.8	0.3	19.3
^k^ 6	Elevated lactate, 2-OH-Butyrate Elevated 3-OH-Butyrate > Acetoacetate	14.3	0.54	26.5
^k^ 7	Massively elevated lactate and moderately dicarboxylic and 3-hydroxybutyric acids	29.9	0.28	9.2 *
8 ^h^	NA	NA	NA	NA
9 ^h^	NA	10.4	0.38	27.4
10 ^h^	NA	11.5	0.73	15.7
11	NA	5.7	0.07	81.1
^k^ 12	Elevated lactate, 2-OH-Butyrate Elevated 3-OH-Butyrate > Acetoacetate	9.5	0.1	95.5
13	Massively elevated lactate and ketones and Krebs metabolites	14.8	0.17	87
14	NA	5.6	0.06	93.2
15	NA	3.2	0.1	31.9
^k^ 16	NA	8.2	0.23	35.5

* The lactate:pyruvate ratio was calculated from the same sample. ^h^ Historical cohort, ^k^ Ketogenic Diet.

**Table 5:** Imaging studies of patients 6,7, 11–13,16.

Patient No.	Globus Pallidum	Dentate Nucleus	Corpus Callosum	Ventriculomegaly	Midbrain and Thalamus
^k^ 16	T2, Flair, DWI high signal, T1 low signal	Normal	Partial agenesis	Mild	Normal
11	T2, Flair, DWI high signal, T1 low signal	T2, Flair, DWI high signal, T1 low signal	Partial agenesis	No	Normal
^k^ 12	T2, Flair, DWI high signal, T1 low signal	Normal	Partial agenesis	Mild	Normal
13	T2, Flair, DWI high signal, T1 low signal	Normal	Partial agenesis	Moderate	Normal
^k^ 6	T2, Flair, DWI high signal, T1 low signal	Normal	Partial agenesis	Mild	Normal
^k^ 7	Normal	T2, Flair, DWI high signal, T1 low signal	Partial agenesis	Mild	T2, Flair, DWI high signal, T1 low signal

^k^ Ketogenic Diet.

## Data Availability

The datasets generated and analyzed during the current study are not publicly available due to patient privacy and confidentiality, but are available from the corresponding author upon reasonable request.

## Supplementary Materials

The following are available online at https://www.mdpi.com/article/10.3390/nu13103523/s1, Table S1. Key clinical and molecular characteristics of patients with dihydrolipoamide dehydrogenase (DLD) deficiency. References [45,46,47,48,49] are referred to in Supplementary Materials.
