# Supplementary material for: The Effects of a Ketogenic Diet on Patients with Dihydrolipoamide Dehydrogenase Deficiency
Source: Nutrients. 2021 Oct 7;13(10):3523. doi: 10.3390/nu13103523 (PMC8540285; doi:10.3390/nu13103523)
Supplement: Supplementary file 1 [file nutrients-13-03523-s001.zip › nutrients-1373313-supplementary.pdf]

Supplementary Table S1. Key clinical and molecular characteristics of patients with dihydrolipoamide dehydrogenase (DLD) deficiency

Pt. No.	Sex	Age at Publication /Death* (D)- (d/w/m/y)	Phenotypic Features					*DLD* Variant	Treatment Modalities	Reference (PMID)
			GER	Liver Disease	Hypoglycemia	DD / ID and Neurological Manifestations	Additional Features			
						Previously reported patients				
1	F	(Infancy)*	NA	NA	NA	Encephalopathy	Emesis	G229C Hom	NA	[1] (14765544)
2	M	16y	NA	NA	NA	Encephalopathy with ataxia, cerebellar and pyramidal signs, intentional tremor	Emesis	G229C Hom	Riboflavin, biotin, coenzyme Q, carnitine	[1] (14765544)
3	M	4y*	NA	NA	Yes	Vegetative state following hypoglycemic episode	NA	G229C Hom	NA	[1] (14765544)
4	F	5y*	NA	Yes	NA	NA	Emesis	G229C Hom	NA	[1] (14765544)
5	F	28m*	NA	NA	Transient neonatal hypoglycemia	DD, hypotonia	NA	Y35X / R460G Comp Het	Dietary restriction of BCAAs, dichloroacetate, thiamine, lipoic acid, carnitine	[2] (8652022), [3] (8968745)
6	M	21m*	NA	NA	NA	Hypotonia, minimal dystonic movements of upper extremities	NA	K72E / P488L Comp Het	Dietary restriction of BCAAs	[4] (3769994) [5] (8506365)
7	M	5.5y*	NA	NA	Yes	DD, seizures	Progressive optic atrophy	G136del / E375K Comp Het	Bicarbonate	[6] (9540846)
8	M	32m*	NA	NA	NA	Truncal hypotonia, limb rigidity and choreoathetoid movements after birth	Mild cortical atrophy, hypertrophic cardiomyopathy	R482G Hom	NA	[7] (1640293) [8] (15712224)

9	M	30m*	NA	NA	NA	Truncal hypotonia, limb rigidity and choreoathetoid movements after birth	Mild cortical atrophy	R482G Hom	Continuous enteral nutrition, bicarbonate	[7] (1640293) [8] (15712224)
10	M	20m*	NA	NA	NA	Truncal hypotonia, limb rigidity and choreoathetoid movements after birth	Mild cortical atrophy, severe growth retardation	R482G Hom	NA	[7] (1640293) [8] (15712224)
11	M	10w	NA	NA	NA	Progressive hypotonia	NA	M361V / E375K Comp Het	High fat, low protein diet, MCT oil, sodium dichloroacetate	[9] (11687750)
12	F	14y	NA	Yes	Yes	Learning disability, episodic encephalopathy, Leigh syndrome	Changes involving BG and CC splenium	I353T / G136del Comp Het	High carbohydrate diet, RRT	[10] (23290025)
13	M	8y	NA	NA	NA	Normal IQ, mild ataxia attention deficit disorder, motor incoordination, severe hypotonia, weakness, impaired vision	NA	Y35X / G229C Comp Het	NA	[11] (9934985)
14	M	5y	NA	Yes	NA	Normal IQ, motor incoordination, moderate hypotonia, weakness	NA	Y35X / G229C Comp Het	NA	[11] (9934985)
15	F	30y	NA	NA	NA	Normal development	Recurrent emesis	G229C Hom	NA	[11] (9934985) [12] (21200621)
16	M	NA	NA	NA	NA	Normal development	Recurrent emesis, exertional fatigue	G229C Hom	NA	[11] (9934985) [12] (21200621)
17	F	NA	NA	NA	NA	Normal development		G229C Hom	NA	[11] (9934985) [12] (21200621)

18	M	NA	NA	NA	NA	Normal development	Exertional fatigue	G229C Hom	NA	[11] (9934985) [12] (21200621)
19	F	13y	NA	NA	NA	Normal development	Myoglobinuria, recurrent emesis	G229C Hom	NA	[11] (9934985) [12] (21200621)
20	M	5y	NA	NA	NA	Normal development	Recurrent emesis	G229C Hom	NA	[11] (9934985) [13] (9161958)
21	M	3y*	NA	Yes	NA	Normal development	Emesis. Died of sepsis	G229C Hom	Dichloroacetate, thiamine, carnitine	[11] (9934985) [13] (9161958)
22	F	19y	NA	NA	NA	Normal development	Exertional fatigue	G229C Hom	NA	[11] (9934985)
23	M	4y	NA	Yes	NA	Severe peripheral neuropathy, episodic delirium, normal development	Recurrent emesis	G229C Hom	Thiamine, dicholoroacetate, night feedings via gastrostomy	[11] (9934985)
24	M	36*	NA	NA	NA	NA	NA	G229C Hom	NA	[11] (9934985)
25	F	34y	NA	Yes	NA	NA	Emesis	G229C Hom		[11] (9934985)
26	F	9m	NA	NA	Yes	Severe DD, hypotonia	Microcephaly	D479V Hom	Bicarbonate, dichloroacetate, carnitine, thiamine, coenzyme Q10, riboflavin	[14] (10448086)
27	M	3y	NA	No	Yes	DD, hypotonia, ataxia, stroke-like episode, tetraspasticity	Microcephaly	IVS+1G>A / I393T Comp Het	Carnitine, vitamins and minerals, KD (80% fat), protein restriction	[15] (12925875)
28	M	2d*	NA	Yes	NA	NA	NA	**	NA	[16] (23478190)
29	M	2d*	NA	Yes	NA	NA	NA	**	NA	[16] (23478190)
30	M	39y	NA	Yes	NA	NA	Reye-like syndrome	G229C Hom	Riboflavin	[16] (23478190)
31	M	31y	NA	Yes	NA	NA	Reye-like syndrome	G229C Hom	Riboflavin	[16] (23478190)

32	M	21y	NA	Yes	NA	NA	Reye-like syndrome	G229C Hom	Riboflavin	[16] (23478190)
33	M	8.5y	NA	Yes	Yes	Recurrent encephalopathy	Abnormalities of BG and frontal lobes on brain MRI	I47T / G229C Comp Het	NA	[17] (16770810)
34	M	3y	NA	NA	NA	Hypotonia, ptosis, weakness	Microcephaly***	I47T / E375K Comp Het	NA	[17] (16770810)
35	M	19y	NA	NA	NA	NA	Progressive exertional fatigue	I40Lfs*4 / G461E	Riboflavin	[18] (25251739)
36	F	32y	NA	NA	NA	NA	Bilateral ptosis, exercise-induced weakness	I480M Hom	KD, thiamine	[19] (20652410)
37	M	4y	NA	Yes	Yes	Persistent hypotonia	NA	G229C Hom	IV glucose	[20] (16601893)
38	M	8m	No	No	No	No	(Asymptomatic)	G229C Hom	NA	[20] (16601893)
						Current publication				
P1	F	* 37m	No	No	No	Severe DD		D479V Hom	Started Ketocal at 2y	-
P2	F	*29m	No	No	Repeated	Severe DD		D479V Hom	Bicarbonate in crisis	-
P3	M	* 3d	No	No	Yes	NA		D479V Hom	Fluids	-
P4	M	*4d	No	No	Yes	NA		D479V Hom	Fluids	-
P5	F	*3m	No	No	Yes	Severe DD		D479V Hom	Fluids	-
P6	M	4.9y	No	During crisis	Yes	Severe DD		D479V Hom	Partial KD & Bicarbonate	-

P7	M	4.1y	Severe	During crisis	Yes	Severe DD	D479V Hom	Partial KD & Bicarbonate	-
P8	M	*3d	No	No	No	NA	D479V Hom	-	-
P9	F	16y	No	Mild during crisis	No	Severe DD	D479V Hom	Canola oil	-
P10	M	14.2y	No	During crisis	During crisis	Severe DD	D479V Hom	-	-
P11	M	4.3y	No	No	During crisis	Moderate to severe DD	D479V Hom	Bicarbonate	-
P12	F	3.2y	No	No	During crisis	Moderate to severe DD	D479V Hom	Partial KD & Bicarbonate	-
P13	M	3.5y	No	No	No	Severe DD	D479V Hom	Bicarbonate	-
P14	F	1.5y	No	No	During crisis	Moderate to severe DD	D479V Hom	Bicarbonate	-
P15	F	1.5y	No	No	During crisis	Moderate to severe DD	D479V Hom	Bicarbonate	-
P16	M	2.8y	No	No	No	Severe DD	D479V Hom	Partial KD & Bicarbonate	-

BCAAs, branched-chain amino acids; BG, basal ganglia; CC, corpus callosum; Comp Het, compound heterozygous; DD, developmental delay; GER, gastroesophageal reflux; Hom, homozygous; ID, intellectual disability; KD, ketogenic diet; MCT, medium chain triglycerides; NA, not available; PMD, psychomotor delay; RRT, renal replacement therapy. *Age of death. **These two infants were siblings of patients no. 24-26, so most probably harbored the homozygous G229C variant as well. ***This patient also had pulmonary arteriovenous malformation attributable to Hereditary Hemorrhagic Telangiectasia due to a maternally inherited 47 amino acid deletion in the *ENG* gene.

References

[1] Hong YS, et al. Identification of a common mutation (Gly194Cys) in both Arab Moslem and Ashkenazi Jewish patients with dihydrolipoamide dehydrogenase (E3) deficiency: possible beneficial effect of vitamin therapy. J Inherit Metab Dis 2003;26(8):816-8. PMID 14765544.

[2] Craigen WJ. Leigh disease with deficiency of lipoamide dehydrogenase: Treatment failure with dichroloacetate. Pediatr Neurol 1996;14:69-71. PMID 8652022.

[3] Hong YS, et al. Identification of two mutations in a compound heterozygous child with dihydrolipoamide dehydrogenase deficiency. Hum Mol Genet 1996;5(12):1925-30. PMID 8968745.

[4] Sakaguchi Y, et al. Dihydrolipoyl dehydrogenase deficiency: a therapeutic trial with branched-chain amino acid restriction. Eur J Pediatr 1986;145(4):271-4. PMID 3769994.

[5] Liu TC, et al. Identification of two missense mutations in a dihydrolipoamide dehydrogenase-deficient patient. *Proc Natl Acad Sci USA*. 1993;90(June):5186-5190. PMID 8506365.

[6] Hong YS, et al. Deficiency of dihydrolipoamide dehydrogenase due to two mutant alleles (E340K and G101del). Analysis of a family and prenatal testing. Biochim Biophys Acta 1997;1362(2-3):160-8. PMID 9540846.

[7] Bonnefont JP, et al. Alpha-ketoglutarate dehydrogenase deficiency presenting as congenital lactic acidosis. J Pediatr 1992;121:255-258. PMID 1640293.

[8] Odièvre MH, et al. A novel mutation in the dihydrolipoamide dehydrogenase E3 subunit gene (DLD) resulting in an atypical form of alpha-ketoglutarate dehydrogenase deficiency. Hum Mutat 2005;25(3):323-4. PMID 15712224.

[9] Cerna L, et al. Novel mutations in a boy with dihydrolipoamide dehydrogenase deficiency. Med Sci Monit 2001;7(6):1319-25. PMID 11687750.

[10] Quinonez SC, et al. Leigh syndrome in a girl with a novel DLD mutation causing E3 deficiency. Pediatr Neurol 2013;48(1):67-72. PMID 23290025.

[11] Shaag A, et al. Molecular basis of lipoamide dehydrogenase deficiency in Ashkenazi Jews. Am J Med Genet 1999;82(2):177-82. PMID 9934985.

[12] Elpeleg O, et al. Recurrent, familial Reye-like syndrome with a new complex amino and organic aciduria. Eur J Pediatr 1990;149(10):709-12. PMID 2120061.

[13] Aptowitzer I, et al. Liver disease in the Ashkenazi-Jewish lipoamide dehydrogenase deficiency. J Pediatr Gastroenterol Nutr 1997;24(5):599-601. PMID 9161958.

[14] Shany E. et al. Lipoamide dehydrogenase deficiency due to a novel mutation in the interface domain. Biochem Biophys Res Commun 1999;262:163-6. PMID 10448086.

[15] Grafokou O, et al. Leigh syndrome due to a compound heterozygousity of dihydrolipoamide dehydrogenase gene mutations: Description of the first E3 splice site mutation. Eur J Pediatr 2003;162(10):714-8. PMID 12925875.

[16] Brassier A, et al. Dihydrolipoamide dehydrogenase deficiency: a still overlooked cause of recurrent acute liver faliure and Reye-like syndrome. Mol Genet Metab 2013;109(1):28-32. PMID 23478190.

[17] Cameron JM, et al. Novel mutations in dihydrolipoamide dehydrogenase deficiency in two cousins with borderline-normal PDH complex activity. Am J Med Genet A 2006;140(14):1542-52. PMID 16770810.

[18] Carrozzo R, et al. Riboflavin responsive mitochondrial myopathy is a new phenotype of dihydrolipoamide dehydrogenase deficiency. The chaperon-like effect of vitamin B12. Mitochondrion 2014;18:49-57. PMID 25251739.

[19] Quintana E, et al. Dihydrolipoamide dehydrogenase (DLD) deficiency in a Spanish patient with mypathic presentation due to a new mutation in the interface domain. J Inherit Metab Dis 2010;33 Suppl 3;S315-9. PMID 20652410.

[20] Sansaricq C, et al. Biochemical and molecular diagnosis of lipoamide dehydrogenase deficiency in a North American Ashkenazi Jewish family. J Inherit Metab Dis 2006;29(1):203-4. PMID 16601893.
